# An Inclusive Offline Learning Platform Integrating Gesture Recognition and Local AI Models

**DOI:** 10.3390/biomimetics10100693

**Published:** 2025-10-14

**Authors:** Marius-Valentin Drăgoi, Ionuț Nisipeanu, Roxana-Adriana Puiu, Florentina-Geanina Tache, Teodora-Mihaela Spiridon-Mocioacă, Alexandru Hank, Cozmin Cristoiu

**Affiliations:** 1 Faculty of Industrial Engineering and Robotics, National University of Science and Technology POLITEHNICA Bucharest, 060042 Bucharest, Romania; florentina.tache@upb.ro (F.-G.T.); teodora.spiridon@upb.ro (T.-M.S.-M.); cozmin.cristoiu@upb.ro (C.C.); 2 Faculty of Engineering in Foreign Languages, National University of Science and Technology POLITEHNICA Bucharest, 060042 Bucharest, Romania; ionut.nisipeanu@stud.fils.upb.ro; 3 National Research and Development Institute for Gas Turbines COMOTI, 061126 Bucharest, Romania; alexandru.hank@comoti.ro

**Keywords:** hands-free interaction, human-centered AI, human factors in computing, gesture-based human–computer interaction, accessible user interfaces

## Abstract

This paper introduces a gesture-controlled conversational interface driven by a local AI model, aimed at improving accessibility and facilitating hands-free interaction within digital environments. The technology utilizes real-time hand gesture recognition via a typical laptop camera and connects with a local AI engine to produce customized learning materials. Users can peruse educational documents, obtain topic summaries, and generate automated quizzes with intuitive gestures, including lateral finger movements, a two-finger gesture, or an open palm, without the need for conventional input devices. Upon selection of a file, the AI model analyzes its whole content, producing a structured summary and a multiple-choice assessment, both of which are immediately saved for subsequent inspection. A unified set of gestures facilitates seamless navigating within the user interface and the opened documents. The system underwent testing with university students and faculty (*n* = 31), utilizing assessment measures such as gesture detection accuracy, command-response latency, and user satisfaction. The findings demonstrate that the system offers a seamless, hands-free user experience with significant potential for usage in accessibility, human–computer interaction, and intelligent interface design. This work advances the creation of multimodal AI-driven educational aids, providing a pragmatic framework for gesture-based document navigation and intelligent content enhancement.

## 1. Introduction

The use of interaction modes that are both natural and intuitive is becoming an increasingly important component in the design of intelligent human–computer interfaces. Touchless interaction, particularly via hand gesture detection, has emerged as a potential route for boosting usability and accessibility in consumer and assistive technologies [1,2]. This change has occurred because of the growth of AI-powered systems and embedded sensors. In the meanwhile, language models have shown impressive skills in processing and creating information in natural language, which has enabled applications in the fields of education, healthcare, and human support systems [3].

Accurate hand tracking with just consumer-grade cameras has been made possible by recent improvements in real-time computer vision, which are driven by frameworks like OpenCV and MediaPipe [4,5,6,7,8]. Thanks to these resources, new multimodal interfaces are possible, allowing for the development of dynamic, context-aware systems that integrate AI reasoning with gesture recognition.

People with disabilities who have trouble using conventional computer input methods might benefit greatly from gesture-controlled systems like the one shown here. Assistive and interactive technologies that rely on gestures can be incredibly helpful for users who have trouble speaking (such as aphasia or post-stroke conditions), have motor limitations that make it hard to use a mouse or keyboard (such as cerebral palsy or muscular dystrophy), or are temporarily unable to move around freely [9,10]. With the suggested system, users no longer need to rely on voice or fine motor control to access AI-assisted learning—even in physically limited settings—thanks to its inclusive design [11].

Gesture detection is a game-changer in human–computer interaction (HCI), allowing touchless interfaces in a wide range of applications. There has been some research on using gesture-based technologies to enhance interactive learning in educational contexts [12]. As an example, Wang et al. [13] presents research on the use of AI for hand gesture detection in VR settings, drawing attention to the technology’s promise as an immersive learning tool.

For real-time applications, there has also been investigation into integrating gesture detection with AI models. To prove that real-time gesture-controlled interfaces are feasible, Sen et al. [14] built a deep learning-based hand gesture detection system that uses CNNs and vision transformers (ViT) to control desktop programs.

The versatility of AI in interpreting complicated gesture inputs was shown by Zeng et al. [15] in their introduction of GestureGPT, a framework that uses big language models to interpret free-form hand gestures, in the field of smart home management and online video streaming.

In addition, gesture-based learning systems (GBLSs) have been making waves in the field of instructional technology. An analysis by GlobalSpec Insights [16] highlights the ways in which GBLSs facilitate active learning by letting students participate with course content via physical gestures. Also, research into the creation of machine learning algorithm-controlled grippers for use in the classroom has been conducted. To provide a more natural interface for managing robotic devices, TheSTEMpedia [17] showcases an example project that uses computer vision and machine learning to understand certain hand gestures in real-time.

The use of radar and wearable sensors to improve interaction capabilities has been investigated in recent advances in gesture recognition. As an example, Yang et al. [18] presented a hand gesture recognition system that uses frequency-shift keying (FSK) radar sensors. This system achieves excellent accuracy at different distances, which is great for applications that need to work well in many types of environments. Wearable technology can provide natural control interfaces, and research on smart gloves with many sensors to record moving hands for use in real-time applications is another example of this [19].

Researchers have looked at using gesture recognition in classrooms to help students of all abilities study together. An excellent illustration of the significance of gesture-based systems in raising student involvement in the classroom is the work of Chen et al. [20], who suggested a continuous recognition algorithm to capture instructors’ dynamic hand signals to aid students with attention problems. In addition, Torres et al. [21] showed that web-based architecture integrating AR and gesture recognition for industrial training scenarios might promote interactive learning experiences via the integration of gesture recognition with AR.

All these studies show that people are very much into the idea of using AI and gesture recognition together to provide instructional tools that everyone can use. For users with accessibility issues, there is a need for more research into systems that integrate these technologies to enable hands-free navigation and engagement with instructional material [22].

In the context of accessible, offline, and hands-free learning environments, the integration of gesture-based control with language-model-powered tools has not been thoroughly investigated [23].

This study presents a gesture-controlled interface that eliminates the need for conventional input devices by using a local AI model and real-time hand gesture detection to help users browse, summarize, and evaluate material. A university environment was used to assess the proposed system’s usability, accuracy in gesture detection, and interaction latency. The findings show that sensor-based educational technologies that combine computer vision and natural language processing have great promises for creating inclusive learning environments.

Recent research has shown that the influence of AI in education is contingent not just on computational correctness but also on institutional support, transparency, and explainability. Choi and Kim [24], for example, showed how explainable AI and data visualization might help people understand finances better by making algorithmic findings understandable to those who are not tech-savvy. Wang et al. [25] also looked at a lot of evidence that AI-based tools can make customization and cooperation better, but they also pointed out that there are still not enough real-world tests. These results inspire our current work, which seeks to provide both technical and educational benefits using an offline, privacy-preserving gesture-AI platform.

## 2. Materials and Methods

**Figure 1:**
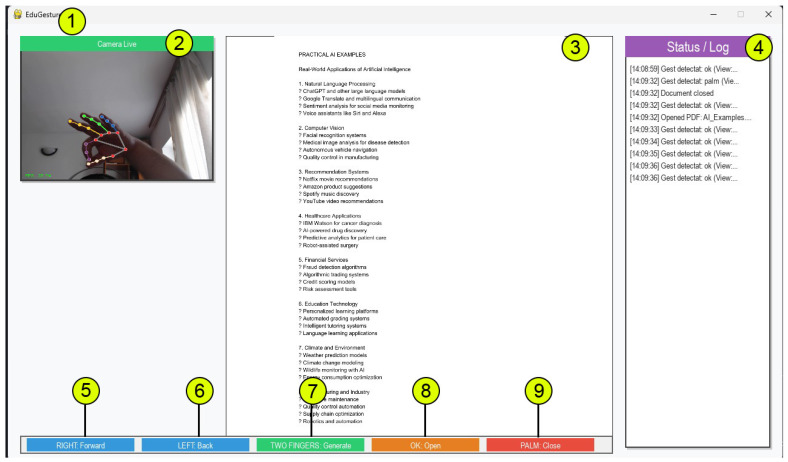
Desktop application that uses a local AI model to create summary and personalized test for specific PDFs, and it is managed by hand gesture: 1. Application interface. 2. Live laptop camera feed. 3. The opened PDF that was selected. 4. Window log of the application. 5. Right sign—to go forward. 6. Left sign—to go back. 7. Two fingers—to generate summary and test using an AI model. 8. OK—to open. 9. Palm—close.

The implemented system has been tested in real time as can be seen in Figure 1. In the next sections, the implementation and testing part is explained.

From 5 to 9 in Figure 1, all the gestures are used to navigate through the folder that contains PDFs files, or to navigate through a selected PDF file.

**Figure 2:**
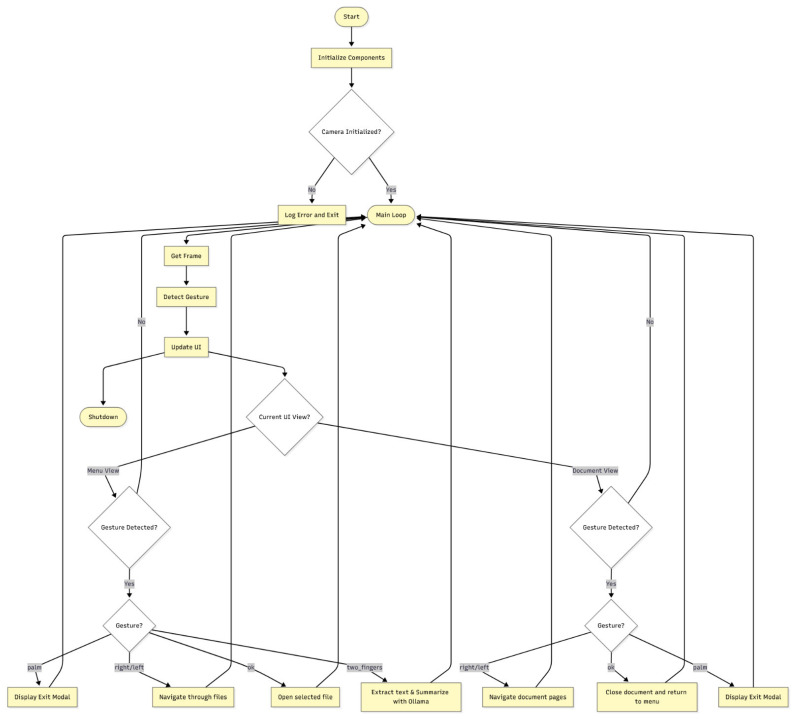
The activity diagram of the proposed system.

The software activity diagram of the system is shown in Figure 2.

### 2.1. The Technology Used

The proposed system is built using a modern Python 3.10 technology stack that combines real-time computer vision, local AI inference, and interactive graphical interfaces to deliver an educational experience controlled by hand gestures. At a high level, the project integrates:

1. Real-time gesture detection using MediaPipe and OpenCV—two widely adopted libraries for video processing and image analysis.
2. Automated generation of educational content (summaries and quizzes) through a local LLM (Large Language Model), accessed via the Ollama API.
3. PDF handling and rendering powered by Python libraries such as PyPDF2, pdf2image, Pillow, and the external Poppler utility for high-fidelity PDF page conversion.
4. A modern graphical user interface (GUI), developed in pygame, which displays camera feed, file list, document pages, and visual logs in real time.

This setup makes the system fully standalone, cross-platform (primarily targeting Windows), and capable of working offline, without relying on cloud services—an ideal solution for educational settings or demonstrations where privacy, responsiveness, and local processing are key.

### 2.2. Methods

#### 2.2.1. Hand Gesture Detection

The proposed system employs MediaPipe and OpenCV from Python to enable real-time hand gesture detection through the user’s webcam. MediaPipe, developed by Google, provides a high-precision hand tracking solution that identifies 21 hand landmarks per frame, even under challenging lighting and movement conditions. These landmarks are extracted from each video frame captured by OpenCV, which handles the live video feed, frame capture, and basic image operations.

Once the hand landmarks are obtained, the system uses geometric rules and vector calculations to classify five gestures: “right”, “left”, “two fingers”, “OK”, and “palm”. For example, it checks whether specific fingers are extended or bent, the relative position of fingertips to the wrist, and directional vectors to determine pointing direction. To ensure smooth interaction, a cooldown mechanism prevents repeated detection of the same gesture in rapid succession. When a gesture is recognized with sufficient confidence, it is logged and translated into a corresponding UI action—such as file navigation, opening a document, or triggering AI-based generation.

This combination of MediaPipe’s hand tracking accuracy and OpenCV’s flexible video processing allows the proposed system to function seamlessly and responsively using only a standard webcam, without requiring additional hardware or depth sensors.

#### 2.2.2. Local LLM

This system uses a local LLM to automatically generate educational summaries and multiple-choice quizzes based on the content of uploaded PDF files. This model is integrated via the Ollama platform [26], which provides a streamlined way to run LLMs locally, without relying on external cloud services. This setup offers significant advantages: low-latency responses, full data privacy, and the ability to function entirely offline [27,28].

Ollama supports downloading and running models like llama3.2:1b, a CPU-optimized model ideal for devices without a dedicated GPU. The presented system leverages this model to analyze the contents of a document and generate a structured summary (highlighting key concepts, definitions, and main ideas) along with a custom multiple-choice quiz. Each quiz question includes three answer options, with the correct one clearly marked, facilitating self-assessment or review.

To enable this feature, users must install Ollama locally by downloading it from the Ollama website and running the command ollama pull llama3.2:1b to fetch the required model. Once Ollama is up and running (ollama serve), the system communicates with it via a local REST API, sending extracted text and receiving AI-generated content in return. This integration makes the system highly flexible and well-suited for a wide range of educational scenarios.

#### 2.2.3. PDF Handling and Rendering

The PDF handling and rendering functionality in this system is implemented using a combination of Python libraries including PyPDF2, pdf2image, Pillow, and Poppler utility. These tools work together to support both text extraction and visual rendering of PDF documents for interaction within the app.

When a PDF file is opened, PyPDF2 is used to parse its structure and extract the raw text content from all pages. This extracted text can then be passed to the local LLM via Ollama for summary and quiz generation. For visual display of PDF pages within the GUI, the app uses pdf2image, which converts each page into a high-resolution image using Poppler (a PDF rendering backend). The resulting images are processed with Pillow for optional resizing and then transformed into OpenCV-compatible arrays for integration with the pygame-based interface.

To ensure cross-platform compatibility—especially for Windows—the proposed system includes logic to detect or manually set the path to the Poppler binary. This allows users to simply add PDF files to the Lessons/folder and navigate or view them with smooth page rendering, even offline. Additionally, when the LLM generates summaries and quizzes, the system uses the fpdf2 library to create properly formatted PDF output.

#### 2.2.4. System GUI

The GUI in the proposed system is built entirely using pygame, a lightweight multimedia library for Python that allows real-time rendering of images, text, and interactive elements. This modern interface provides users with a clear and intuitive way to interact with the system using only hand gestures, without relying on keyboard or mouse input.

The GUI is structured into several visual components (see Figure 1):

- A live camera preview panel (showing gesture detection in real time);
- A file list panel that displays all documents in the Lessons/folder with indicators for file type (PDF or TXT);
- A document viewer, capable of rendering either PDF page images or paginated text content;
- A log/status panel, which shows color-coded messages based on user actions and system events;
- A gesture legend bar at the bottom, which updates dynamically to remind the user of available gesture commands.

The interface also includes a modal confirmation dialog (e.g., when exiting), designed with visual overlays and stylized highlights. Also, the GUI leverages advanced layout logic and color schemes to create a desktop app-like experience, responsive and optimized for educational interaction.

### 2.3. System Implementation

The implementation of the system adheres to a modular, object-oriented programming (OOP) paradigm, using Python 3.10+. The source code is structured into dedicated modules, each encapsulated within its own file and class-based abstraction (e.g., GestureDetector, PDFHandler, OllamaConnector, UIManager, and MainController as the main controller). This design fosters separation of concerns, simplifies debugging, and enables easier future extensions or replacements of individual components.

From a software architecture perspective, the system follows an event-driven model where the primary input events are hand gestures, and their interpretation triggers context-aware actions depending on the current UI state (menu or document). The main application loop handles these events synchronously, while time-intensive operations such as AI generation are dispatched to separate threads using Python’s built-in threading module to prevent UI blocking and ensure responsiveness.

Each class was designed with a single responsibility principle (SRP) in mind:

- GestureDetector handles only image processing and gesture classification;
- PDFHandler abstracts file operations and rendering logic;
- UIManager handles visual rendering, layout, and user feedback;
- OllamaConnector communicates with the AI backend in a stateless manner;
- Stats in utils.py tracks usage analytics in structured CSV logs.

To ensure portability and clarity, configuration parameters (such as the Ollama host or model) are loaded using the python-dotenv package from a .env file, enabling environment-specific overrides without code changes. File paths, fonts, and rendering settings are explicitly defined and validated, making the system robust to filesystem or platform inconsistencies.

The codebase applies defensive programming techniques such as the following:

- Explicit exception handling (try-except) around all external calls (e.g., camera capture, file I/O, AI inference);
- Fallback routines (e.g., default image when PDF rendering fails);
- Logging of all user actions and system-level events for traceability.

Function and method names follow Pythonic naming conventions, with consistent docstrings for maintainability. The entire UI layer operates at ~60 FPS, and graphical updates are tightly controlled to minimize CPU usage, using Pygame’s clock.tick() mechanism.

Lastly, the system avoids external state dependencies: all processing is performed locally, no cloud services are required, and all logs and outputs (e.g., generated tests) are saved in structured formats for reproducibility. This ensures that the implemented system can be deployed and executed fully offline, making it suitable for accessibility-focused educational contexts where connectivity is limited or privacy is paramount.

### 2.4. System Control

**Figure 3:**
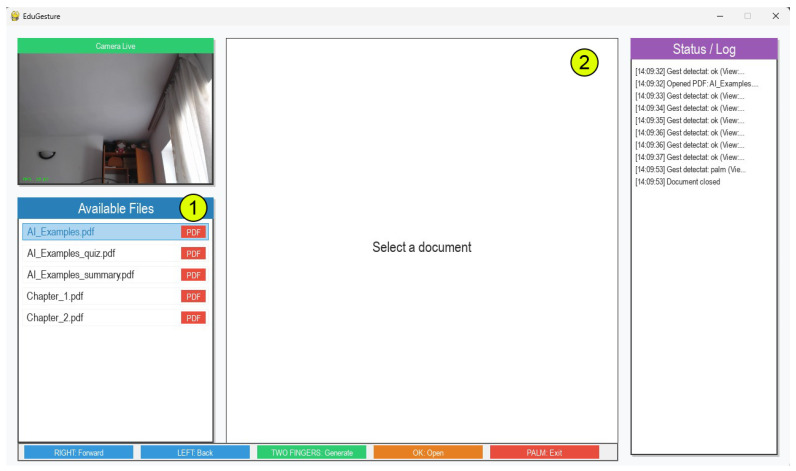
System menu with available PDF files and focus on the first file from folder: 1. Folder with available PDF files. 2. View of the selected file via hand gestures.

The control logic of the proposed system is centralized in the main.py module, which acts as the orchestrator of all system components. The system follows a state-driven architecture, where the interface can be in one of two main states: menu (file selection view, see Figure 3) or document (active file view). The current state determines how incoming gestures are interpreted and routed to appropriate handlers.

System control is based on a main event loop, which captures video frames from the webcam, processes them for gesture recognition, and dynamically updates the user interface using Pygame. Detected gestures are passed through a gesture dispatching mechanism that maps them to actions depending on the current state, and the examples of which are as follows:

- A right gesture navigates to the next file when in the menu, or to the next page when in document view.
- A palm gesture either closes the current document or triggers the exit confirmation modal.

Asynchronous tasks, such as interactions with the local LLM via Ollama, are launched in separate threads, allowing the system to remain responsive while the AI processes content in the background. Thread safety is maintained by isolating shared state variables and updating them only through controlled transitions.

The system includes built-in checks for system health and error handling. For instance, if the camera feed fails, the system attempts automatic reinitialization, and logs are updated in real-time to reflect the system status. A shutdown flag (shutdown_event) is monitored continuously, ensuring clean termination of all resources—camera, files, UI, and gesture logs—when the application is closed.

This centralized, state-aware control system ensures consistency, responsiveness, and robustness, enabling a smooth user experience even under variable input conditions or hardware limitations.

## 3. Results

The proposed system was evaluated with 31 participants, who tested the system in both menu and document navigation modes (see Table 1). The evaluation focused on three core metrics: gesture recognition accuracy, command-response latency, and user-perceived usability.

To ensure consistent and comparable results across all participants, the tests were conducted using the same educational PDF document, approximately 6 pages long (around 2500 words). This standardized approach ensured uniform interaction patterns and allowed meaningful analysis of system behavior and AI processing times. The reported average time for AI-based summary and quiz generation (12.86 s) refers specifically to this shared document.

Across all test sessions, the system achieved an average gesture recognition accuracy of 86.7%. The most reliably detected gestures were palm (98%) and OK (96%), while the left and right pointing gestures showed slightly lower precision (approx. 90%) due to more subtle directional angles. Misclassification occurred mostly in low-light conditions or when the hand was partially occluded.

The results also show noticeable variability across participants, with gesture recognition accuracy ranging from a high of 96.3% to a low of 64.7%. This spread suggests that individual factors such as gesture execution style, hand positioning, and personal familiarity with the interface may influence recognition performance. A similar dispersion was observed in average response times, which varied from 492 ms to 1554 ms across participants. Such differences indicate that both user behavior and system–environment interactions can have a measurable impact on overall responsiveness, highlighting the importance of optimizing recognition robustness for a wide range of usage conditions. These performance variations provide useful context for interpreting the mean latency and AI generation times reported in the following paragraph.

The average system response time from gesture detection to interface action was measured at ~872 ms, including gesture recognition, command dispatching, and UI update. The generation of AI content (summary + quiz) via Ollama averaged 12.86 s, depending on document length and system load. These values were acceptable for real-time interaction in a learning context and did not interrupt user flow.

Qualitative feedback was collected via post-test questionnaires using a 5-point Likert scale. Users rated the system’s ease of use at 4.03/5, and around 80% expressed strong interest in applying it for inclusive or hands-free educational scenarios. Notably, participants highlighted the benefit of interacting with documents without touching the keyboard or mouse, especially for accessibility use cases.

During the evaluation, several practical scenarios emerged:

- Touchless navigation of lecture slides or textbooks in teaching contexts.
- AI-generated quizzes used for self-assessment after reading.
- Use by individuals with temporary or permanent motor limitations.

These results suggest that the proposed system performs reliably in real-time and can be effectively deployed in educational environments requiring minimal physical interaction.

## 4. Discussion

The evaluation results demonstrate that the proposed system achieves reliable performance in real-time gesture-controlled navigation and AI-assisted content generation. With an average gesture recognition accuracy of 86.7%, the system performed consistently across the 31 participants, despite differences in hand shape, movement style, and environmental conditions. The palm and OK gestures showed the highest recognition rates (98% and 95%, respectively), confirming their robustness and clear visual features. In contrast, left and right pointing gestures, while still maintaining a solid 88% precision, were more prone to misclassification in low-light settings or when executed at subtle angles. This highlights the importance of gesture selection in system design, as some movements are inherently easier for vision-based models to detect reliably.

The measured average command-response latency of ~872 ms is within an acceptable range for interactive applications. This value encompasses the entire pipeline—from frame capture and gesture detection to command execution and UI update—and was found to be short enough to maintain a smooth, responsive user experience. For AI content generation, the 12.86 s average processing time using a local large language model represents a reasonable trade-off between computational complexity and offline operation. Given that the generation step produces both a structured summary and a quiz, this latency is unlikely to disrupt the user’s learning flow, especially in scenarios where these resources are meant for later review.

The variation between the minimum and maximum response times (492 ms vs. 1554 ms) indicates that user-specific factors and environmental conditions can significantly affect system performance. This spread suggests that while the average latency is acceptable, ensuring consistent responsiveness across diverse contexts remains a challenge. Similarly, AI content generation times varied between 10.3 s and 15.9 s, highlighting the need for further optimization to guarantee predictable performance in real-time usage scenarios.

User feedback further supports the system’s usability, with an average ease-of-use score of 4.03/5 and around 80% of participants expressing strong interest in adopting the system for hands-free or accessibility-focused contexts. Qualitative comments highlighted its potential for enabling educators to navigate lecture materials without manual input, as well as for supporting individuals with temporary or permanent motor impairments. This aligns with prior research indicating that gesture-based interfaces can enhance engagement and reduce barriers in educational environments.

When compared to related systems described in the literature, the system offers several advantages. Unlike many cloud-dependent gesture recognition tools, it operates entirely offline, ensuring privacy and enabling deployment in resource-constrained settings such as classrooms without reliable internet access. The integration of a local LLM via Ollama distinguishes it from gesture-only systems by providing on-demand, personalized content augmentation. This multimodal approach—combining natural interaction with AI-driven summarization and assessment—has the potential to increase both learning efficiency and accessibility.

From a technological standpoint, the ability to navigate, summarize, and generate outputs without any physical contact introduces new possibilities for both instructors and learners. In classroom scenarios, educators can move through lecture materials hands-free, maintaining engagement with students and reducing reliance on peripheral devices. For users with motor impairments or temporary mobility restrictions, the system offers an inclusive pathway to access, process, and review digital content autonomously. By lowering interaction barriers and combining them with AI-driven personalization, the system can contribute to improved accessibility, learner autonomy, and overall participation in educational activities. These benefits align with broader goals in inclusive education and universal design for learning (UDL), where technology serves to accommodate diverse needs without compromising the quality of instruction.

The evaluation also reveals areas for improvement. The lower recognition rates for lateral pointing gestures suggest that the detection algorithm could benefit from more robust directional feature extraction, potentially through a hybrid approach that combines geometric rules with lightweight machine learning classifiers. Lighting variability remains a notable challenge, and future iterations could incorporate adaptive brightness and contrast normalization to improve robustness. On the AI side, although 12.86 s generation time is acceptable for offline operation, model optimization or incremental summarization strategies could further reduce latency, making the system more responsive for rapid review scenarios.

The results confirm that the system successfully addresses its primary goals of enabling hands-free navigation through educational content and generating useful learning materials locally, without reliance on external infrastructure. Its combination of real-time gesture control, offline AI processing, and user-oriented design makes it a promising tool for inclusive education, particularly in contexts where accessibility, privacy, and adaptability are critical. With targeted refinements in gesture recognition and performance optimization, the system could serve as a scalable, deployable platform for gesture-based interaction in both formal and informal learning environments.

Compared with previous gesture-based educational systems, such as StarC (Chen et al. [29]) and the continuous recognition framework proposed by Chen et al. [20], our platform focuses on static, lightweight gestures compatible with consumer webcams and fully offline processing. While dynamic-gesture systems can achieve richer interaction, they often require additional sensors or training data, which limits accessibility. Future development may integrate adaptive lighting normalization and hybrid geometric–machine-learning models to improve robustness, following approaches inspired by Chen et al. [20].

Furthermore, in line with Choi and Kim [24] and Wang et al. [25], our results underline that sustainable AI-based learning systems must combine technical inclusivity with educational and institutional frameworks that promote transparency and long-term adoption.

## 5. Conclusions

The proposed system demonstrates that real-time gesture recognition, when integrated with local AI-powered content generation, can deliver a practical, inclusive, and privacy-preserving interaction tool. The system succeeds in bridging the gap between touchless human–computer interaction and personalized educational resource creation, allowing users to navigate documents and generate tailored learning materials entirely offline. By combining MediaPipe-based gesture tracking with a locally hosted large language model, the system avoids the privacy and latency concerns typically associated with cloud-dependent solutions, making it suitable for deployment in environments with limited or no internet access.

The evaluation with 31 participants confirmed that the platform provides an accessible and efficient alternative to traditional input methods. The high recognition rates for key gestures such as palm and OK indicate that the gesture set was effectively chosen for robustness and usability. Although some gestures exhibited slightly lower accuracy under suboptimal conditions, the overall performance was sufficient to maintain smooth interaction. The AI generation component reliably produced structured summaries and quizzes in a timeframe that supports uninterrupted learning sessions, while the unified user interface ensured that navigation and content creation were integrated seamlessly.

From a broader perspective, the system contributes to the development of multimodal interactive technologies that prioritize inclusivity without sacrificing functionality. Its design directly addresses the needs of users with temporary or permanent motor limitations, while also offering practical benefits in teaching contexts where hands-free control can improve efficiency—such as when delivering lectures or conducting live demonstrations. The system’s offline operation and modular architecture further enhance its adaptability for varied institutional, home-based, or field-learning scenarios.

The proposed system offers a solid foundation for further innovation. Future work could explore expanding the gesture vocabulary, integrating adaptive recognition models for different lighting and background conditions, and introducing real-time feedback to guide gesture performance. Optimizing the local AI model or enabling incremental content generation could reduce latency and improve responsiveness for interactive teaching situations. Moreover, extending support for additional document formats and multilingual content generation would broaden the system’s applicability across diverse educational settings.

While the system achieved promising results, certain constraints must be acknowledged. The gesture recognition module, though robust for clear and well-lit scenarios, remains sensitive to low-light environments, partial occlusions, and variations in camera placement. Additionally, the gesture set was intentionally limited to five commands to ensure simplicity, which may restrict interaction possibilities in more complex workflows. The AI generation time, although acceptable for most educational uses, could be a limiting factor in highly dynamic settings requiring rapid, repeated content creation. Furthermore, the current evaluation was conducted on a small, controlled group of participants, which may not fully capture performance variations across broader demographics or real-world classroom conditions.

Planned developments aim to address these limitations and extend the capabilities of the system. Enhancing the gesture recognition pipeline with adaptive image preprocessing and lightweight machine learning classifiers could improve accuracy in variable lighting and background conditions. The gesture vocabulary could be expanded to include contextual commands, enabling more complex interactions without compromising usability.

On the AI side, implementing model optimization, caching strategies, or incremental processing could reduce generation times and support more interactive use cases. Broader usability studies across different age groups, educational levels, and accessibility needs will be essential for refining the system’s ergonomics and adoption potential. Finally, integrating the system with complementary interactive technologies—such as virtual classrooms, e-learning platforms, or augmented reality environments—could position it as a versatile tool within the evolving ecosystem of digital interaction.

In addition, future evaluations should involve a larger and more diverse participant sample and be conducted in real educational environments rather than exclusively under controlled laboratory conditions. Such studies would provide deeper insights into real-world usability, long-term adoption potential, and the system’s impact on teaching and learning outcomes.

## Figures and Tables

**Table 1:** Testing results.

Participant	Gesture Accuracy (%)	AVG Response Time (ms)	AI Generation Time (s)	Ease of Use (1–5)
1	87.5	752	13.2	4
2	81.0	913	12.8	3
3	93.0	861	12.7	5
4	96.3	1554	13.0	2
5	77.8	653	15.9	5
6	71.7	1339	14.4	3
7	87.5	905	12.6	4
8	81.0	620	10.3	5
9	64.7	788	12.6	4
10	87.5	492	14.2	4
11	90.2	715	12.9	5
12	85.9	971	12.8	4
13	88.6	1033	13.1	3
14	83.4	702	13.0	4
15	95.1	863	11.4	5
16	86.8	746	12.6	4
17	89.3	804	12.8	5
18	92.5	1346	13.0	5
19	80.7	735	12.9	3
20	95.2	511	12.7	5
21	84.2	845	13.0	2
22	91.0	980	11.7	5
23	88.1	649	12.8	4
24	83.8	831	12.9	4
25	90.4	834	11.9	5
26	82.7	735	12.5	3
27	89.6	1036	12.9	4
28	92.1	835	13.3	5
29	85.6	800	13.1	4
30	90.8	718	12.8	2
31	93.7	1432	13.0	5
Total AVG	86.7	871.9	12.86	4

## Data Availability

The original contributions presented in this study are included in the article. Further inquiries can be directed to the corresponding author.
